# *In situ* expression of ERG protein in the context of tumor heterogeneity identifies prostate cancer patients with inferior prognosis

**DOI:** 10.1002/1878-0261.13225

**Published:** 2022-06-18

**Authors:** Susanne G. Kidd, Mari Bogaard, Kristina T. Carm, Anne Cathrine Bakken, Aase M. V. Maltau, Marthe Løvf, Ragnhild A. Lothe, Karol Axcrona, Ulrika Axcrona, Rolf I. Skotheim

**Affiliations:** ^1^ Department of Molecular Oncology, Institute for Cancer Research Oslo University Hospital–*Radiumhospitalet* Norway; ^2^ Institute for Clinical Medicine, Faculty of Medicine University of Oslo Norway; ^3^ Department of Pathology Oslo University Hospital–*Radiumhospitalet* Norway; ^4^ Department of Urology Akershus University Hospital Lørenskog Norway; ^5^ Department of Informatics, Faculty of Mathematics and Natural Sciences University of Oslo Norway

**Keywords:** biomarker, ERG, ETS, heterogeneity, prognosis, prostate cancer

## Abstract

Prognostic biomarkers for prostate cancer are needed to improve prediction of disease course and guide treatment decisions. However, biomarker development is complicated by the common multifocality and heterogeneity of the disease. We aimed to determine the prognostic value of candidate biomarkers transcriptional regulator ERG and related ETS family genes, while considering tumor heterogeneity. In a multisampled, prospective, and treatment‐naïve radical prostatectomy cohort from one tertiary center (2010–2012, median follow‐up 8.1 years), we analyzed ERG protein (480 patients; 2047 tissue cores), and RNA of several ETS genes in a subcohort (165 patients; 778 fresh‐frozen tissue samples). Intra‐ and interfocal heterogeneity was identified in 29% and 33% (ERG protein) and 39% and 27% (ETS RNA) of patients, respectively. ERG protein and ETS RNA was identified exclusively in a nonindex tumor in 31% and 32% of patients, respectively. ERG protein demonstrated independent prognostic value in predicting biochemical (*P* = 0.04) and clinical recurrence (*P* = 0.004) and appeared to have greatest prognostic value for patients with Grade Groups 4*–*5. In conclusion, when heterogeneity is considered, ERG protein is a robust prognostic biomarker for prostate cancer.

## Graphical abstract

The prognostic relevance of the most common mutations in prostate cancer, involving *ERG* and other ETS genes, is still unclear. Utilizing a multisampled biobank, we show that ERG expression is heterogeneous both within and between malignant foci in the same patient. Despite significant heterogeneity, we find that ERG is a biomarker for poor prognosis for patients with localized prostate cancer.

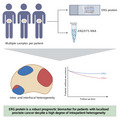

**Abbreviations**
BCR: biochemical recurrence
cDNA: complementary DNA
CI: confidence interval
CR: clinical recurrence
C_T_
: cycle threshold
FFPE: formalin‐fixed paraffin‐embedded
HR: hazard ratio
HGPIN: high‐grade prostatic intraepithelial neoplasia
IDC‐P: intraductal carcinoma of the prostate
ISUP: International Society of Urological Pathology
IQR: interquartile range
PCR: polymerase chain reaction
PSA: prostate‐specific antigen
pT‐stage: pathological tumor stage
Q3: third quartile
REMARK: Reporting recommendations for tumor marker prognostic studies
RP: radical prostatectomy
RT: reverse transcription
TMA: tissue microarray

## 1 Introduction

There is an unmet need for prognostic biomarkers in prostate cancer to aid in predicting clinical outcome of primary disease and improve the precision of treatment decisions [1]. Currently, none of the available molecular biomarker tests are recommended for routine use, but may be relevant in certain clinical settings [2]. Prostate cancer is commonly multifocal [3] with a high degree of intra‐ and interfocal heterogeneity [4, 5, 6], which complicates the development of clinically useful biomarkers.

One group of potential biomarkers are fusion genes involving oncogenic members of the ETS family of transcription factors, including *ERG*, *ETV1*, *ETV4*, and *FLI1* [7]. These fusion genes are the most frequent molecular aberrations in primary prostate cancer, with *TMPRSS2*‐*ERG* being the most common (present in approximately 50% of patients [8]) [7, 9]. Accordingly, they have been proposed as part of a molecular subtyping framework [7]. These ETS genes are not expressed in normal prostate epithelial cells, and aberrations in them are considered to be early driver events [10, 11]. The ETS family is characterized by a common ETS DNA‐binding domain with a helix‐turn‐helix motif, which is essential for DNA recognition and binding, and thus their role as transcription factors [12]. *ERG* and other ETS factors are involved in a variety of normal physiological processes, for example, angiogenesis and vascular homeostasis, through regulation of genes specific to endothelial cells (*e.g*., VE‐cadherin) and the Wnt/β‐catenin pathway [10, 13]. In cancer, oncogenic ETS proteins, including *ERG*, *ETV1*, and *ETV4*, have been found to bind a common set of genomic regions that potentially regulate processes such as differentiation, proliferation, and angiogenesis [14]. It has also been shown that ETS proteins can function as substitutes in RAS/MAPK signaling [14].

Intra‐ and interfocal heterogeneity of aberrations in ETS genes has been demonstrated [4, 6, 15]. *TMPRSS2*‐*ERG* has been associated with adverse histopathological features, for example, cribriform growth pattern [16], intraductal carcinoma of the prostate (IDC‐P) [17], and stromal changes [18]. However, the prognostic value of aberrations in *ERG* [19] and other ETS genes is still inconclusive, which is best explained by the lack of sufficiently large studies accounting for the common multifocality and heterogeneity of prostate cancer.

Recently, persistently elevated prostate‐specific antigen (PSA) after radical prostatectomy (RP) was demonstrated to be associated with adverse prognosis and advanced disease [20], and potentially applicable in risk stratification [21]. Patients with undetectable PSA may represent a patient subgroup with superior prognosis; however, even some of these patients experience relapse, warranting additional biomarkers for risk stratification. Persistent PSA has, to our knowledge, not been considered in previous studies assessing the prognostic implications of aberrations in *ERG* and other ETS genes.

In the present study, we have determined the prognostic value of *ERG* and other ETS genes in a large, multisampled, prospective cohort of primary prostate cancer patients with long‐term follow‐up, while considering multifocality, heterogeneity, and persistent PSA.

## 2 Materials and methods

### 2.1 Study population and prostate cancer biobank

The prospective cohort includes 571 prostate cancer patients treated with RP at Oslo University Hospital–*Radiumhospitalet* between 2010 and 2012, as previously described [4, 5, 22]. The biobank includes formalin‐fixed paraffin‐embedded (FFPE) and fresh‐frozen tissue samples. Patients who received radiation and/or hormone therapy prior to RP, had metastatic disease at time of surgery, or with unavailable tissue slides were excluded, leaving a total of 515 patients in the present study (Fig. S1).

The study was undertaken with the understanding and written consent of each patient. The study methodologies conformed to the standards set by the Declaration of Helsinki and were approved by the Regional Ethics Committee South‐East Norway (number 2013/595).

The study is reported according to the Reporting recommendations for tumor marker prognostic studies (REMARK; Table S1).

### 2.2 Follow‐up data

Follow‐up data consist of PSA measurements, type and reason for any additional treatments for prostate cancer (adjuvant, salvage, and palliative treatments), and, if applicable, date and cause of death. A dedicated research nurse oversees and collects all relevant follow‐up data. PSA values have been collected through correspondence with general practitioners, review of hospital records, and from Fürst Medical Laboratories (Oslo, Norway). The median follow‐up time for all patients included in the study was 8.1 years [interquartile range (IQR): 7.2–8.8 years], with a median of 16 (IQR: 12–22) PSA measurements collected for each patient. For patients without biochemical recurrence (BCR) and without persistently PSA, the median follow‐up time was 8.0 years (IQR: 7.1–8.6 years), with a median of 14 PSA measurements (IQR: 10–18) collected. BCR was defined as a postoperative PSA level ≥ 0.20 ng·mL^−1^ in two consecutive blood samples collected at least 1 week apart, where the first of these dates was used as the time point for BCR. A persistent PSA was defined as PSA level of ≥ 0.10 ng·mL^−1^ at 4–8 weeks after RP. Clinical recurrence (CR) was defined as verified recurrence of prostate cancer and included local recurrence, lymph node, and/or distant metastasis. Data on CR were obtained through review of medical records. Date and cause of death were obtained from the population‐based Norwegian Cause of Death Registry.

### 2.3 Histopathological assessment

Histopathological re‐evaluation of RP specimens was performed according to the 2014 International Society of Urological Pathology (ISUP) Modified Gleason system [23] by two investigators (MB and UA). For analyses using fresh‐frozen tissue, the area surrounding where the tissue sample was collected was used to evaluate the Gleason score and the histopathological features: reactive stroma, minor high‐grade pattern 5, and cribriform pattern (invasive cribriform carcinoma and/or IDC‐P). Reactive stroma was evaluated as present or absent, and based solely on morphology [24]. Minor high‐grade pattern 5 was defined as a Gleason grade 5 component present in < 5% of the tumor specimen in Grade Group 2 and 3 tumors. The presence of cribriform pattern was evaluated based on morphology [25].

For all RP specimens, multifocality was assessed and it was determined from which focus each tissue sample was collected. Tumors were defined as different foci when clearly separated by at least 2–4 mm and showing different tissue morphology. The index tumor was defined as the focus with the highest pathological tumor (pT)‐stage. In cases with multiple foci with the same pT‐stage, the focus with the highest Gleason score, or in cases with two foci with the same Gleason score, the largest focus (in diameter), was defined as the index tumor.

### 2.4 Tissue microarray construction

Construction of tissue microarray (TMA) blocks was performed using FFPE tissue from the 506 patients with available FFPE tissue blocks, and the Tissue Arrayer (Beecher Instruments, Silver Spring, MD, USA) instrument, with 1.0 mm tissue cores and 80–120 tissue cores per recipient paraffin block. The Microtome HM355S (Thermo Fisher Scientific, Waltham, MA, USA) was used to cut 4 μm sections, and regions of interest were verified on hematoxylin–eosin stained sections.

### 2.5 *In situ* ERG protein analysis by immunohistochemistry

From the TMAs, 480 patients had at least one malignant sample that could be evaluated for *in situ* ERG protein expression. In total, 2047 tissue cores (1447 malignant, 600 benign) could be evaluated, of which 1–11 malignant tissue cores (median: 3 tissue cores) and 0–6 benign tissue cores (median: 1 tissue core) from each patient. Multiple tissue cores from the same malignant tumor focus were available for 312 patients and from multiple foci from 156 patients.

*In situ* protein expression of ERG was assessed with immunohistochemistry on the TMAs using the fully automated Ventana Benchmark Ultra system, with anti‐ERG monoclonal antibody EPR3864 (Roche Tissue Diagnostics, Tucson, AZ, USA) and Ventana UltraView Universal DAB Detection kit (Roche Tissue Diagnostics). Tissue from tonsils, liver, pancreas and appendix were used as controls for ERG immunohistochemistry. Endothelial cells were used as an internal positive control. ERG protein expression was visually scored and evaluated as positive or negative as previously described [26, 27]. A tissue core was classified as positive if any percentage of malignant cells showed positive nuclear staining.

### 2.6 ETS RNA analysis

Fresh‐frozen tissue was included from a subcohort of patients (*N* = 165). A total of 778 fresh‐frozen tissue samples (359 malignant and 419 benign) were analyzed, with 0–6 malignant samples (median: 2 samples) and 0–7 benign samples (median: 2 samples) from each patient. Multiple samples from the same malignant focus were available for 94 patients and from multiple foci for 56 patients.

RNA was isolated from fresh‐frozen tissue samples with the AllPrep DNA/RNA/miRNA Universal kit (Qiagen, Venlo, Netherlands). Complementary DNA (cDNA) was generated by reverse transcription (RT) of total RNA using either the High Capacity cDNA Reverse Transcription kit (Thermo Fisher Scientific) or SMARTer™ RACE cDNA Amplification kit (Clontech, Mountain View, CA, USA) according to the manufacturer's protocols.

Semi‐quantitative RNA expression levels of four ETS genes (*ERG*, *ETV1*, *ETV4*, and *FLI1*) and one reference gene (*ABL1*) were determined with real‐time RT polymerase chain reaction (PCR), in a reaction volume of 10 μL, using TaqMan Universal Master Mix II, with UNG (Thermo Fisher Scientific) and TaqMan Gene Expression assays (Thermo Fisher Scientific): Hs01554630_m1 (*ERG*), Hs00231877_m1 (*ETV1*), Hs00944562_m1 (*ETV4*), Hs00956709_m1 (*FLI1*), and Hs01104728_m1 (*ABL1*). All samples were run in triplicates on an ABI 7900HT Fast Real‐Time PCR System (Thermo Fisher Scientific), with 10 ng cDNA input in each reaction. *ABL1* was selected as a reference gene based on its stable expression in prostate cells, as shown in previous studies [4, 28] and in in‐house RNA sequencing data from 88 tissue samples from prostate cancer patients [29].

Median cycle threshold (*C*_T_) values for all sample triplicates were used in subsequent analyses. Expression of the ETS genes was normalized to the reference gene *ABL1* using the standard curve method. The ratio between the median relative quantities of ETS and *ABL1* in benign samples was used as a calibrator. Thresholds for overexpression were set using a formula for extreme outliers, Q3 + 3 * IQR (Q3, third quartile of log_2_‐transformed fold changes of the ETS gene in benign samples; IQR of log_2_‐transformed fold changes of the ETS gene in benign samples). Samples with *C*_T_ medians > 35 were considered as having no expression and thus assigned to the ‘no overexpression’ group.

*TMPRSS2*‐*ERG* fusion transcripts were detected by RT‐PCR with 50 ng cDNA included in each reaction, using the HotStar Taq DNA Polymerase kit (Qiagen). Primers were designed with the primer3 software (Whitehead Institute for Biomedical Research, Cambridge, MA, USA) and had the following sequences: GGGGAGCGCCGCCTGGAG (*TMPRSS2*, forward primer) and CCCACCATCTTCCCGCCTTTG (*ERG*, reverse primer). Gel electrophoresis with a 2% agarose gel was run at 200 V for 30 min. A Universal Hood II (Bio‐Rad Laboratories, Hercules, CA, USA) was used for visualization. Photographs were generated using the image lab software (version 2.0.1, build 18, Bio‐Rad Laboratories) and visually inspected to determine *TMPRSS2*‐*ERG* fusion status. For samples with inconclusive results, an additional RT‐PCR and electrophoresis were performed.

The sequence identities of selected RT‐PCR products (15 samples from 10 patients) were determined with Sanger sequencing, using the illustra ExoProStar 1‐step kit (Cytiva, Marlborough, MA, USA), BigDye Terminator v1.1 Cycler Sequencing kit (Thermo Fisher Scientific), and BigDye XTerminator Purification kit (Thermo Fisher Scientific), followed by capillary electrophoresis on an AB3730 DNA Analyzer (Thermo Fisher Scientific) with POP‐7 polymer (Thermo Fisher Scientific). Sequencing Analysis Software v5.3.1 (Thermo Fisher Scientific) was used to assess the sequences, and exons were annotated with Ensembl release 97.

### 2.7 Statistical analysis

The χ^2^ test of independence or Fisher's exact test were used to assess associations between categorical variables, whereas the Wilcoxon rank‐sum test was used to compare continuous variables. In time‐to‐event analyses, BCR and CR were used as endpoints. Patients with a persistent PSA and/or who received adjuvant treatment were excluded from time‐to‐event analyses (*N* = 63). Patients without BCR or CR were censored at the date of their latest known PSA measurement. Kaplan–Meier plots were generated and log‐rank tests applied to compare time to BCR or CR. Kaplan–Meier curves were truncated when the number at risk in each group was less than five. Univariable and multivariable Cox regression analyses were performed to obtain hazard ratios (HR) with 95% confidence intervals (CI). The Schoenfeld test was applied to assess whether the proportional hazards assumption was met. An interaction term was tested in Cox regression analyses where relevant, and the likelihood‐ratio test was used to compare statistical models. A *P*‐value of 0.05 was used as threshold for statistical significance. All analyses were performed using r (version 4.1.1; The R Foundation, Vienna, Austria) and rstudio (version 1.4.1717; R Studio Inc., Boston, MA, USA). Time‐to‐event analyses were performed using the ‘survival’ (version 3.2–10) and ‘survminer’ (version 0.4.9) packages.

## 3 Results

### 3.1 ERG protein expression and heterogeneity

**Fig. 1:**
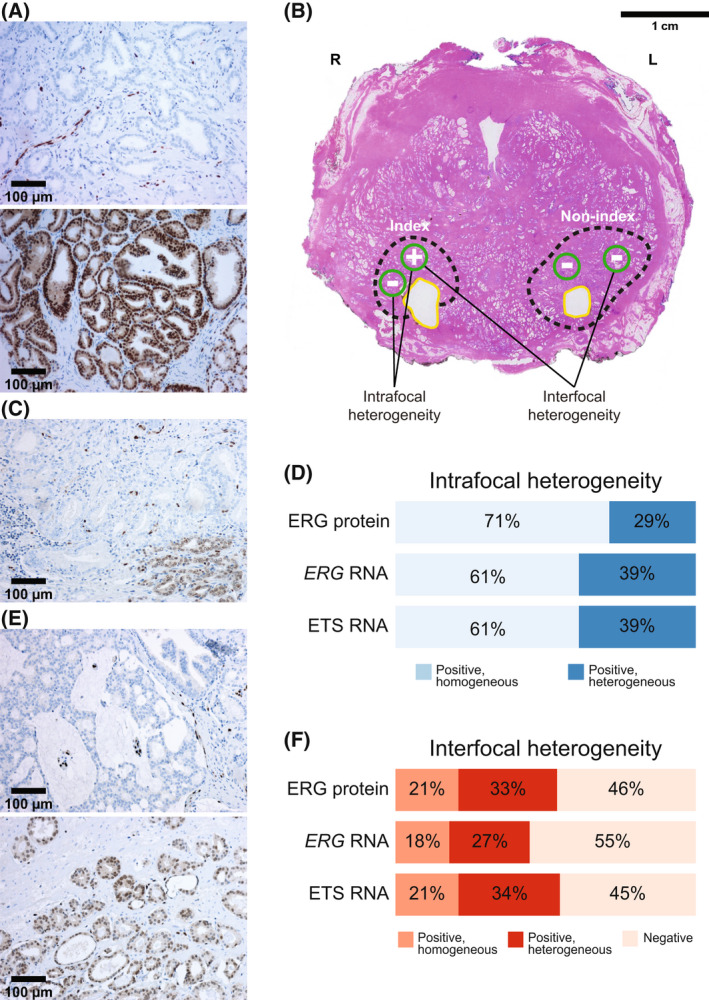
Heterogeneity in expression of ERG protein and ETS RNA in prostate cancer. (A) Representative images of positive (top) and negative (bottom) staining for ERG protein on tissue microarrays (10x magnification; scale bar = 100 μm). Endothelial cells were used as an internal positive control. A patient was classified as ERG‐positive if at least one tissue core had any percentage of malignant cells with positive nuclear staining. In total, 51% (244/480) of patients, and 43% (625/1447) of malignant samples, were ERG‐positive. (B) Whole‐mount prostatectomy specimen illustrates two malignant tumor foci (dashed lines). R and L indicate right and left sides, respectively. Green circles illustrate areas sampled from formalin‐fixed paraffin‐embedded tissue for protein analyses, whereas areas marked in yellow represent where fresh‐frozen tissue samples were collected. From the fresh‐frozen tissue, RNA was isolated and analyzed. Intrafocal heterogeneity was defined as the same focus having at least one positive and one negative sample, either for ERG protein or ETS RNA overexpression. Interfocal heterogeneity was defined as the patient having at least one positive and one negative malignant focus and was assessed in patients with samples from at least two malignant foci. Scale bar = 1 cm. (C) Intrafocal heterogeneity of ERG protein expression within a single tissue core (10x magnification; scale bar = 100 μm). (D) Intrafocal heterogeneity of ERG protein expression and RNA overexpression of *ERG* and ETS genes combined. Intrafocal heterogeneity was identified in 29% (50/170) of patients for ERG protein, and in 39% of patients for both *ERG* RNA (19/49) and ETS overexpression (24/61). (E) Interfocal heterogeneity of ERG protein expression in two different malignant foci from the same patient. The top picture shows a negative malignant focus, and the bottom a positive malignant focus (10x magnification; scale bar = 100 μm). (F) Interfocal heterogeneity of ERG protein expression and RNA overexpression of *ERG* and ETS genes combined. Interfocal heterogeneity was identified in 33% (52/156) of patients for ERG protein, 27% (15/56) for *ERG* RNA, and 34% (19/56) for ETS RNA overexpression. Scale bars were generated using imagej (version 1.53 K; National Institutes of Health, Bethesda, MD, USA).

ERG protein expression was assessed by immunohistochemistry (Fig. 1A) and identified in at least one malignant sample from 51% (244/480) of patients, in a total of 43% (625/1447) of malignant samples (Tables S2 and S3). No ERG protein expression was detected in benign tissue. A patient was defined as ERG‐positive if one or more malignant samples were positive, and a malignant focus as positive if one or more samples from that focus were positive. Intra‐ and interfocal heterogeneity of ERG protein expression was identified in 29% (50/170) and 33% (52/156) of patients with multiple samples from either the same ERG‐positive focus or different foci, respectively (Fig. 1B–F). Among patients with interfocal heterogeneity, 31% (16/52) were exclusively ERG‐positive in a non‐index focus.

### 3.2 Associations between ERG protein and clinicopathological characteristics

ERG protein was associated with slightly younger age at time of surgery, lower preoperative PSA levels, higher pT‐stages, cribriform pattern, and reactive stroma (Table 1).

**Table 1:** Clinicopathological characteristics of the cohort, stratified by the patient's ERG protein status. The *χ* ^2^ test of independence was used for categorical variables and the Wilcoxon rank‐sum test for continuous variables. Asterisks (*) indicate statistical significance, *P* < 0.05. IQR, interquartile range; *N*, number of patients; pN‐stage, pathological lymph node stage; PSA, prostate‐specific antigen; pT‐stage, pathological tumor stage; RP, radical prostatectomy.

Characteristic	ERG‐positive (*N* = 243; 51%)	ERG‐negative (*N* = 237; 49%)	*P*
Age at time of surgery, median (IQR)	63 (59–66)	65 (61–69)	< 0.001*
Preoperative PSA in ng·mL^−1^, median (IQR)	9.7 (6.6–16.0)	11.0 (8.0–18.0)	0.007*^,^ ^a^
Grade Group for RP‐specimen, *N* (%)			
1–2	130 (53)	111 (47)	0.3
3	62 (26)	71 (30)	
4–5	51 (21)	55 (23)	
Multifocal cancer, *N* (%)	156 (64)	167 (70)	0.2
Positive surgical margins, *N* (%)	38 (16)	40 (17)	0.8
pT‐stage, *N* (%)			
pT2	86 (35)	116 (49)	0.009*
pT3a	124 (51)	97 (41)	
pT3b	33 (14)	23 (10)	
Missing	0 (0)	1 (0.4)	
pN‐stage, *N* (%)			
pN0	53 (22)	64 (27)	0.2^b^
pN1	13 (5)	8 (3)	
pNX	177 (73)	165 (70)	
Cribriform pattern, *N* (%)	148 (61)	112 (47)	0.004*
Reactive stroma, *N* (%)	106 (44)	79 (33)	0.03*
Minor high‐grade pattern 5, *N* (%)			
Present	34 (14)	40 (17)	0.3^c^
Absent	145 (60)	126 (53)	
Not applicable	64 (26)	71 (30)	
Persistent PSA after RP, *N* (%)	20 (8)	18 (8)	0.9

^a^ One patient was excluded as there was no available information about preoperative PSA.

^b^ pN0 *vs*. pN1.

^c^ Absent *vs*. present.

### 3.3 Prognostic value of ERG protein

Twenty percent (83/422) of patients experienced BCR with a median time to BCR of 3.2 years (IQR: 1.8–5.4 years), while 11% (46/422) experienced CR with a median time to treatment start for CR from time of surgery of 5.0 years (IQR: 3.4–7.0 years). Among patients with CR, 13% (6/46) developed metastatic castration‐resistant disease. In total, 9% (40/422) of patients died during follow‐up, whereof two have died of prostate cancer.

**Fig. 2:**
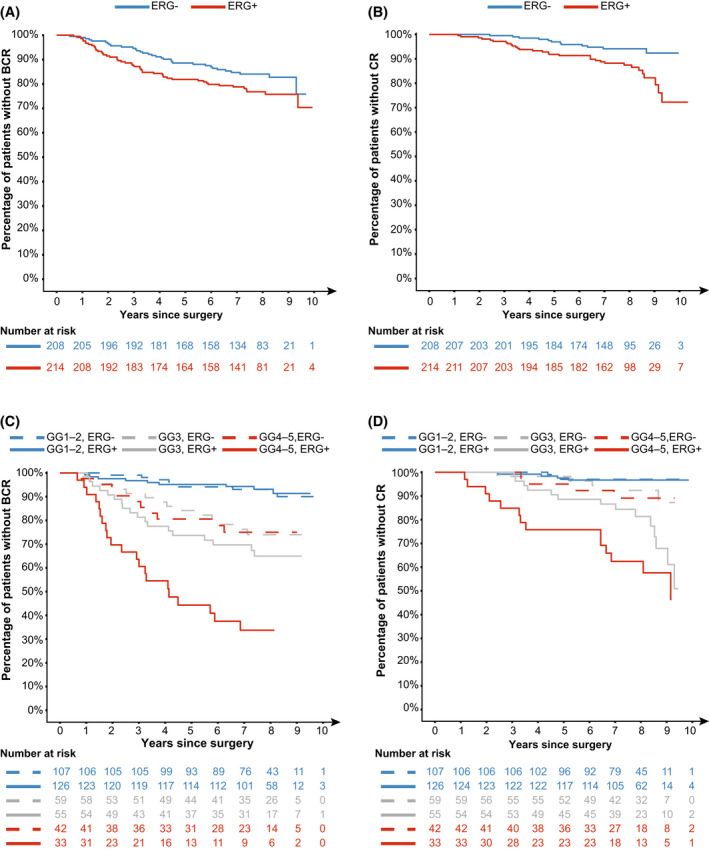
Prognostic value of ERG protein expression. Patients with persistently elevated PSA levels and/or who received adjuvant treatment postradical prostatectomy were excluded from time‐to‐event analyses (*N* = 63). Kaplan–Meier plots were truncated when the number at risk in a specific group was less than five. (A) Fraction of patients without biochemical recurrence, stratified by ERG protein status. There was no statistically significant association between ERG status and biochemical recurrence (log‐rank test, *P* = 0.1). (B) Fraction of patients without clinical recurrence, stratified by ERG protein status. A statistically significant association between ERG status and clinical recurrence was identified (log‐rank test, *P* = 0.008). (C–D) Fraction of patients without (C) biochemical recurrence or (D) clinical recurrence, stratified by ERG status and Grade Group. BCR, biochemical recurrence; CR, clinical recurrence; ERG‐, ERG‐negative; ERG+, ERG‐positive; GG, Grade Group; PSA, prostate‐specific antigen.

To evaluate the association between ERG protein and clinical outcome, time‐to‐event analyses were performed (Fig. 2). ERG protein was significantly associated with CR [HR: 2.35; 95% CI: (1.23–4.48); *P* = 0.01], but the association did not reach significance for BCR [HR: 1.44; 95% CI: (0.93–2.24); *P* = 0.1] in univariable analysis (Table S4). In multivariable analyses, ERG protein was independently associated with both BCR and CR (Table 2). It appeared that the association of ERG protein with prognosis was dependent on Grade Group (Fig. 2C–D). An interaction term between ERG protein and Grade Group was explored, but was neither statistically significant for BCR (likelihood‐ratio test, *P* = 0.06) nor CR (likelihood‐ratio test, *P* = 0.4).

**Table 2:** Expression of ERG protein as a predictor of biochemical and clinical recurrence. Multivariable Cox regression analyses with biochemical or clinical recurrence as endpoints. Patients with persistently elevated PSA levels and/or who received adjuvant treatment postradical prostatectomy were excluded from analyses (*N* = 63). Except for ERG, only variables that were significant in univariable analyses were included in multivariable analyses. The proportional hazards assumption was met for all analyses. One patient was excluded from analyses as there was no information on preoperative PSA. Asterisks (*) indicate statistical significance, *P* < 0.05. Hyphens (−) indicate that the analysis was not performed. CI, confidence interval; HR, hazard ratio; *N*, number of patients; number of events, number of patients experiencing either biochemical or clinical recurrence; pN‐stage, pathological lymph node stage; PSA, prostate‐specific antigen; pT‐stage, pathological tumor stage; RP, radical prostatectomy.

Covariable	Biochemical recurrence (*N* = 421, number of events = 83)		Clinical recurrence (*N* = 422; number of events = 46)	
	HR (95% CI)	*P*	HR (95% CI)	*P*
Preoperative PSA (continuous)	1.01 (0.99*–*1.02)	0.4	–	–
ERG protein expression				
Negative	1.00 (reference)		1.00 (reference)	
Positive	1.62 (1.02*–*2.58)	0.04*	2.65 (1.37*–*5.11)	0.004*
Grade Group for RP‐specimen				
1–2	1.00 (reference)		1.00 (reference)	
3	3.37 (1.87*–*6.10)	< 0.001*	4.72 (1.96*–*11.4)	< 0.001*
4–5	4.85 (2.49*–*9.44)	< 0.001*	6.74 (2.70*–*16.8)	< 0.001*
pT‐stage				
pT2	1.00 (reference)		1.00 (reference)	
pT3a	1.99 (1.09*–*3.63)	0.02*	2.21 (0.94*–*5.22)	0.07
pT3b	3.93 (1.86*–*8.29)	< 0.001*	5.02 (1.89*–*13.3)	0.001*
pN‐stage				
pN0	1.00 (reference)			
pN1	2.92 (1.11*–*7.65)	0.03*	–	–
pNX	1.20 (0.70*–*2.03)	0.5	–	–
Surgical margins				
Negative	1.00 (reference)			
Positive	2.86 (1.63*–*5.00)	< 0.001*	–	–

### 3.4 Expression of ETS genes on the RNA level

**Fig. 3:**
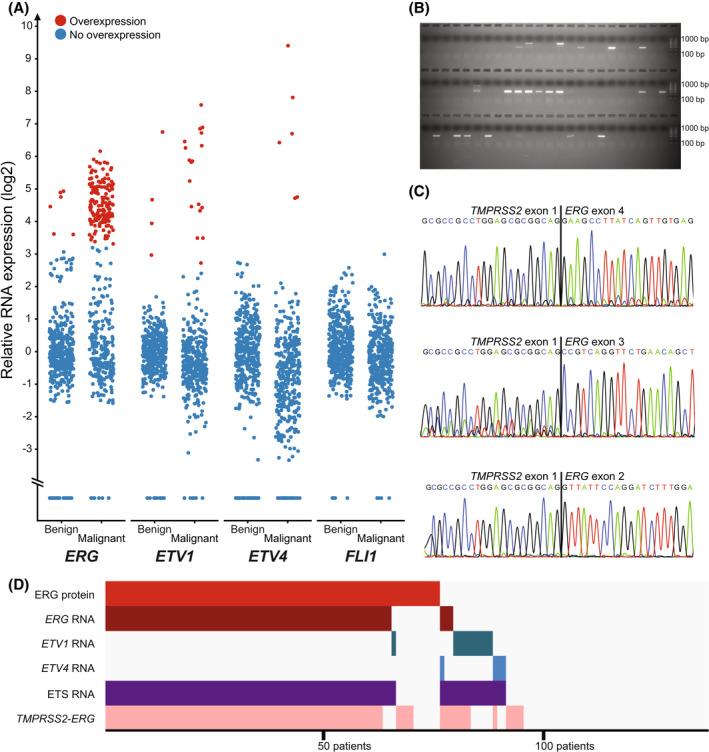
RNA expression of ETS genes and concordance with ERG protein. (A) Relative RNA expression levels of four ETS genes (*ERG*, *ETV1*, *ETV4*, and *FLI1*) in 419 benign and 359 malignant samples from 165 prostate cancer patients, as determined by real‐time RT‐PCR. Individual thresholds for overexpression were calculated for each gene. Twenty patients were excluded from further analysis due to only having benign samples. In total, 48% (70/145), 8% (12/145), and 3% (5/145) of patients had overexpression of *ERG*, *ETV1*, and *ETV4* in at least one malignant sample, respectively. No patients overexpressed *FLI1*. Samples with no expression are shown as data points at the bottom of the plot. (B) An example gel image demonstrating the result of RT‐PCR followed by gel electrophoresis. Samples with visible bands on the gel were scored as positive for *TMPRSS2*‐*ERG* expression. In total, 56% (81/145) of patients had expression of this fusion gene in malignant samples. *TMPRSS2*‐*ERG* was also detected in benign tissue from 31% of patients (50/161) with available benign samples. A 100 bp size marker was included on both ends on all well rows, with the shortest fragment being 100 bp and the longest 1000 bp. (C) Three *TMPRSS2*‐*ERG* fusion breakpoint sequences identified with Sanger sequencing. Sanger sequencing was performed for 15 samples from 10 patients. The sequences correspond to *TMPRSS2* exon 1 (ENST00000332149.10) and *ERG* exon 4, 3, and 2 (ENST00000442448.5). The fusion breakpoint between *TMPRSS2* exon 1 and *ERG* exon 4 was found to be the most common (13 samples). The exons were annotated with Ensembl release 97. (D) Concordance between ERG protein, ETS RNA overexpression, and *TMPRSS2*‐*ERG* RNA expression could be determined for 137 patients where both protein and RNA data were available. Each column represents one patient. Gray indicates that the patient is negative for ERG protein, ETS RNA overexpression, or *TMPRSS2*‐*ERG* RNA. RT‐PCR, reverse transcription polymerase chain reaction.

We investigated the RNA expression levels of several ETS genes (*ERG*, *ETV1*, *ETV4*, and *FLI1)* and their association with prognosis, in a largely overlapping subcohort (*N* = 165, of which at least one malignant sample was available from 145 patients; Fig. 3A). Fusion breakpoint‐spanning analysis was performed to detect *TMPRSS2*‐*ERG* RNA (Fig. 3B–C). Patients were scored as ETS‐positive if at least one malignant sample had overexpression of one or more ETS genes. In total, 59% (85/145) of patients were ETS‐positive, of which 82% (70/85) were positive for *ERG*, 14% (12/85) for *ETV1*, and 6% (5/85) for *ETV4*. Among malignant samples, 46% (165/359) had RNA overexpression of either *ERG* (89%, 141/159), *ETV1* (11%, 18/159), or *ETV4* (1%, 6/159). No samples displayed overexpression of *FLI1*. Overexpression of multiple ETS genes was detected in different malignant foci from 6% (2/31) of ETS‐positive patients with malignant samples from more than one focus. Among these, one patient was positive for both *ERG* and *ETV4*, and the other for *ETV1* and *ETV4*. None had overexpression of multiple ETS genes in the same malignant focus. *TMPRSS2*‐*ERG* was detected in 47% (167/359) of malignant samples, with 56% (81/145) of patients being positive for *TMPRSS2*‐*ERG* in at least one malignant sample (Tables S2 and S5). *ERG* RNA overexpression and *TMPRSS2*‐*ERG* status was concordant in 88% (128/145) of patients, whereas *ERG* RNA and *TMPRSS2*‐*ERG* were concordant with ERG protein in 90% (123/137) and 85% (116/137), respectively (Fig. 3D).

Similar to ERG protein, substantial heterogeneity was identified in RNA expression of both *ERG* and ETS genes combined (Fig. 1D and F). Among patients with more than one sample from the same positive malignant focus, intrafocal heterogeneity was identified in 39% for both ETS (24/61) and *ERG* RNA overexpression (19/49). Interfocal heterogeneity was identified in 34% (19/56) of patients for ETS and 27% (15/56) for *ERG* RNA overexpression, respectively. Among patients with interfocal heterogeneity of ETS expression, 32% (6/19) were exclusively ETS‐positive in a nonindex focus. ETS overexpression was associated with a younger age at time of surgery, higher pT‐stage, and reactive stroma (Table S6).

Among patients positive for *ERG* RNA, 39% (27/70) experienced BCR and 21% (15/70) CR. Similar proportions were identified in patients positive for *ETV1* and/or *ETV4*, where 31% (5/16) experienced BCR and 13% (2/16) CR. There was no significant association between BCR or CR and RNA overexpression of *ERG* or ETS genes combined (Fig. S2 and Table S7).

## 4 Discussion

We demonstrate that ERG protein is an independent prognostic biomarker for prostate cancer patients not receiving adjuvant treatment and with undetectable PSA after RP, and that ERG is of particular importance for Grade Group 4*–*5 patients. In agreement with previous findings, ERG protein was associated with morphological phenotypes associated with poorer prognosis, including reactive stroma, invasive cribriform carcinoma, and IDC‐P [30, 31], which support that ERG protein is a biomarker for aggressive prostate cancer. The present study shows that substantial tumor heterogeneity necessitates analyses of multiple malignant areas to fully appreciate the prognostic impact of ERG protein in prostate cancer.

The extensive molecular heterogeneity in prostate cancer has become more acknowledged in recent years [4, 5, 22, 32]. Still, the identification of clinically useful prognostic biomarkers is limited by the fact that studies seldom consider tumor heterogeneity [4, 22]. In agreement with others, we find substantial intra‐ and interfocal heterogeneity of *ERG* expression on both the protein and RNA levels [6, 33]. Intrafocal heterogeneity may be due to different cell populations within a malignant focus, which could occur if two foci have merged but visually appear as one [34, 35]. Moreover, interfocal heterogeneity supports the idea that different foci are independent tumors without a shared precursor [5]. We found that ERG protein and ETS RNA overexpression occurred exclusively in a nonindex focus in approximately one third of patients, underlining the importance of analyzing multiple malignant foci to accurately determine ERG protein/ETS RNA status, and not just the tumor believed to be the index focus. RNA overexpression of multiple ETS genes in different malignant foci from the same patient further highlights the vast heterogeneity.

The prognostic value of *ERG* aberrations has previously presented with conflicting results [19]. However, the lack of analyses in the context of tumor heterogeneity suggests that such aberrations may have been underestimated. Indeed, we show that ERG protein in at least one malignant sample is associated with poor clinical outcome, in terms of both BCR and CR, independent of known prognostic clinicopathological features, including surgical margin status. These findings suggest that ERG protein drives and promotes the development of metastatic disease. Accordingly, ERG‐negative patients may have a better prognosis.

The association between ERG protein and clinical outcome was studied in patients with undetectable PSA and not receiving adjuvant treatment after RP. This patient group is of particular interest for novel risk stratification, as they are currently considered to have a better prognosis [36], although some will still experience relapse and lethal disease. In our cohort, one fifth of these patients developed BCR and close to 10% CR. In total, 40 patients have died during the follow‐up period, but only two as a result of prostate cancer, which limits the use of overall death and prostate cancer‐specific death as endpoints in survival analysis. Localized prostate cancer has a natural long course of disease, with the median time to development of metastases after BCR being 8 years and another 5 years from metastasis to death [37]. Consequently, a longer follow‐up time would be needed to fully assess the association between ERG and death.

In addition to *ERG*, other ETS genes are believed to be oncogenic [10], and assessment of multiple ETS genes may improve prognostic stratification of patients. We find similar trends for clinical outcome for RNA overexpression of *ERG* and ETS genes combined, suggesting that additional ETS genes should not be disregarded as prognostic biomarkers. However, as aberrations in other ETS genes are less common [7], larger studies analyzing a multisampled cohort are required to fully elucidate the implication of aberrations in additional ETS genes.

Expression of *TMPRSS2*‐*ERG* was identified in benign samples from a notable proportion of patients, and some also had overexpression of ETS genes. ETS aberrations are thought to be early events [10], and *TMPRSS2*‐*ERG* has previously been detected in high‐grade prostatic intraepithelial neoplasia (HGPIN) [38]. As we did not differentiate between HGPIN and benign glands, this could explain our findings of ETS aberrations in benign samples. Furthermore, the tissue collected for RNA analyses could not be evaluated directly, but rather the surrounding tissue, so that malignant cells could be present in the sample. We did not identify ERG protein in benign tissue, even in patients where *TMPRSS2*‐*ERG* was detected. This has also been observed by others and could imply that the RNA methods are more sensitive, in that areas negative for ERG protein may not produce a sufficient amount of protein or a variant that is not recognized by the applied antibody [39]. Another likely explanation, although speculative, is that heterogeneity in *ERG* expression is present even within benign tissue.

The results from analysis of *ERG* RNA expression in malignant samples demonstrated a distinct separation into ‘high’ and ‘low’ groups, which supports the evaluation of ERG protein as either positive or negative. Other studies have also applied this scoring system [26]. However, it would be interesting to assess alternative approaches based on further division of the percentage of ERG‐positive malignant cells to determine whether the prognostic impact differs, although this is likely better suited for assessment on whole tissue sections.

Although beyond the scope of the current study, the prognostic relevance of certain *ERG* variants has gained increasing interest [40]. Multiple *ERG* RNA transcripts isoforms can be expressed, resulting in at least 15 protein variants [12]. Some of these variants may have a higher oncogenic potential and have been associated with more advanced prostate cancer [41]. Accordingly, the prognostic relevance of ERG may differ based on which protein variant is expressed. Taken together, these studies support the need for further investigation of the prognostic value of specific protein variants.

A high concordance between ERG protein, *ERG* RNA overexpression, and *TMPRSS2*‐*ERG* was identified among malignant samples, in line with other studies [42]. The few discrepancies could be due to other fusion partners than *TMPRSS2* or RT‐PCR being a more sensitive method. The antibody used for immunohistochemistry has known cross‐reactivity with FLI1 protein, but this is an unlikely explanation as none of the samples had *FLI1* RNA overexpression. Nonetheless, the high concordance demonstrates that the methods are largely interchangeable for the detection of *ERG* aberrations. As FFPE tissue is routinely collected, immunohistochemistry is the preferred method and could easily be implemented in most laboratories.

Overall, our results support the implementation of ERG protein assessment in the post‐RP setting to determine which patients require closer follow‐up and are potential candidates for adjuvant treatment. Due to the vast heterogeneity, all malignant foci should be evaluated. According to the European Association of Urology guidelines for prostate cancer [36], it is already recommended to state whether multifocality is present in RP specimens, which would likely allow for a simpler implementation of ERG assessment of all malignant foci in daily practice.

The significant heterogeneity of ERG complicates its use as a biomarker in the diagnostic setting; however, a study by Shah *et al*. [43], suggested an approach that adequately detects ERG expression in needle biopsies while also being cost‐effective. Future studies assessing ERG protein in diagnostic needle biopsies are warranted to determine whether it may improve risk stratification and treatment selection.

## 5 Conclusions

*In situ* ERG protein expression is an independent predictor of BCR and CR in prostate cancer patients with undetectable PSA and who did not receive adjuvant treatment after surgery. Significant intra‐ and interfocal heterogeneity of ERG protein expression challenges previous accuracy of ERG status assessment. We conclude that implementation of ERG protein as a prognostic biomarker in the post‐RP setting while considering the vast heterogeneity may aid treatment decisions and improve patient outcomes.

## Conflict of interest

The authors declare no conflict of interest.

## Author contributions

ML, RAL, KA, UA, and RIS were all involved in conception and design. SGK, MB, KTC, ACB, and AVM performed acquisition of data. All authors were involved in analysis and interpretation of data. SGK and MB performed statistical analysis. RAL, KA, UA, and RIS were responsible for obtaining funding. ACB, AVM, and ML were involved in administrative, technical, or material support. ML, RAL, KA, UA, and RIS supervised the study.

6

### Peer Review

The peer review history for this article is available at https://publons.com/publon/10.1002/1878‐0261.13225.

## Supporting information


**Fig. S1.** Flow chart of patient inclusion.
**Fig. S2.** Prognostic relevance of overexpression of *ERG* RNA and ETS genes combined.
**Table S1.** REMARK checklist (Reporting recommendations for tumor marker prognostic studies).
**Table S2.** Clinicopathological and molecular characteristics for all patients. *See separate Excel file*.
**Table S3.** Protein expression of ERG in all evaluable samples. *See separate Excel file*.
**Table S4.** Univariable Cox regression analysis of ERG protein expression with biochemical recurrence and clinical recurrence as endpoints.
**Table S5.** RNA expression of four ETS genes (*ERG, ETV1, ETV4* and *FLI1*) and *TMPRSS2‐ERG. See separate Excel file*.

**Table S6.** Clinicopathological characteristics of the patient subcohort analyzed on the RNA level, stratified by ETS status.
**Table S7.** Univariable Cox regression analysis of ETS RNA overexpression with biochemical recurrence and clinical recurrence as endpoints.

## Data Availability

The majority of the data that support the findings of this study are available from the supporting information published online, and any additional data are available from the corresponding author upon reasonable request.
