# Supplementary material for: In situ expression of ERG protein in the context of tumor heterogeneity identifies prostate cancer patients with inferior prognosis
Source: Mol Oncol. 2022 Jun 18;16(15):2810–22. doi: 10.1002/1878-0261.13225 (PMC9348599; doi:10.1002/1878-0261.13225)
Supplement: Supplementary file 1 — Fig. S1. Flow chart of patient inclusion. Fig. S2. Prognostic relevance of overexpression of ERG RNA and ETS genes combined. Table S1. REMARK checklist (Reporting recommendations for tumor marker prognostic studies). Table S2. Clinicopathological and molecular characteristics for all patients. See separate Excel file. Table S3. Protein expression of ERG in all evaluable samples. See separate Excel file. Table S4. Univariable Cox regression analysis of ERG protein expression with biochemical recurrence and clinical recurrence as endpoints. Table S5. RNA expression of four ETS genes (ERG, ETV1, ETV4 and FLI1) and TMPRSS2‐ERG. See separate Excel file. [file MOL2-16-2810-s002.doc]

# **Supporting information**

for the paper:

***In situ* expression of ERG protein in the context of tumor heterogeneity identifies prostate cancer patients with inferior prognosis**

Susanne G. Kidd^*^, Mari Bogaard^*^, Kristina T. Carm, Anne C. Bakken, Aase V. Maltau, Marthe Løvf, Ragnhild A. Lothe, Karol Axcrona, Ulrika Axcrona^**^, and Rolf I. Skotheim^**,†^

*^*^Shared first authors*

*^**^Shared senior authors*

*^†^Corresponding author*

# Table of contents

Figure S1: Flow chart of patient inclusion. *Page no. 2*

Figure S2: Prognostic relevance of overexpression of *ERG* RNA and ETS genes combined. *Page no. 3*

Table S1: REMARK checklist (Reporting recommendations for tumor marker prognostic studies). *Page no. 4–5*

Table S2: Clinicopathological and molecular characteristics for all patients. *See separate Excel file*

Table S3: Protein expression of ERG in all evaluable samples. *See separate Excel file*

Table S4: Univariable Cox regression analysis of ERG protein expression with biochemical recurrence and clinical recurrence as endpoints. *Page no. 6*

Table S5: RNA expression of four ETS genes (*ERG*, *ETV1*, *ETV4* and *FLI1*) and *TMPRSS2*-*ERG*. *See separate Excel file*

Table S6: Clinicopathological characteristics of the patient subcohort analyzed on the RNA level, stratified by ETS status. *Page no. 7*

Table S7: Univariable Cox regression analysis of ETS RNA overexpression with biochemical recurrence and clinical recurrence as endpoints. *Page no. 8*


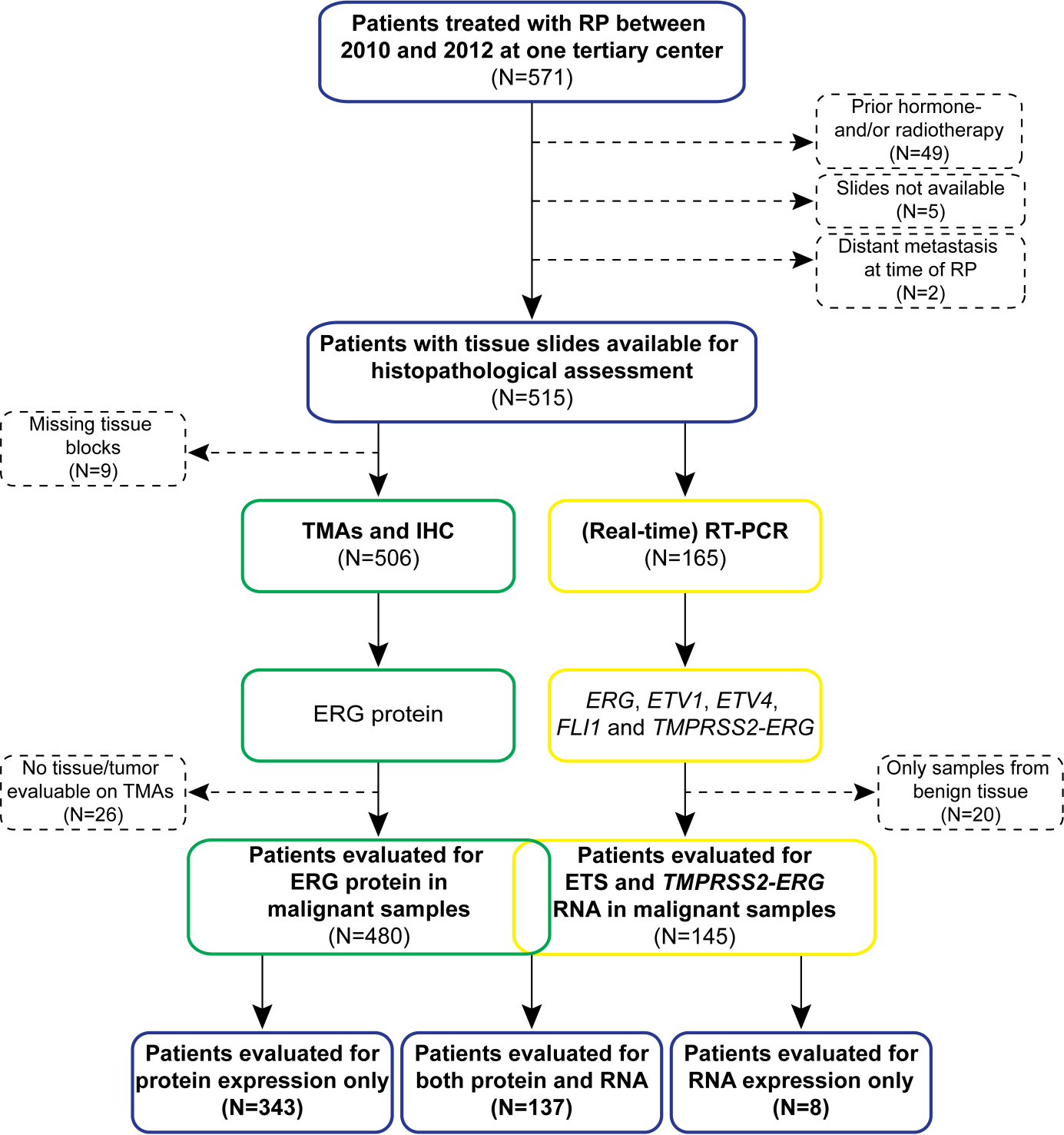


## Figure S1. Flow chart of patient inclusion. Only patients that had not received treatment prior to radical prostatectomy, with available tissue slides, and without distant metastasis at the time of radical prostatectomy were included in the study. Malignant samples from 480 patients could be evaluated for ERG protein status, as determined by immunohistochemistry. Patients analyzed on the RNA level (real-time RT-PCR and RT-PCR) were included consecutively (N=120), in addition to patients selected for having samples from multiple malignant foci (N=45). Overlap between green and yellow boxes indicates that patients were included in both protein and RNA analyses. IHC, immunohistochemistry; RP, radical prostatectomy; RT-PCR, reverse transcription polymerase chain reaction; TMA, tissue microarray.

##


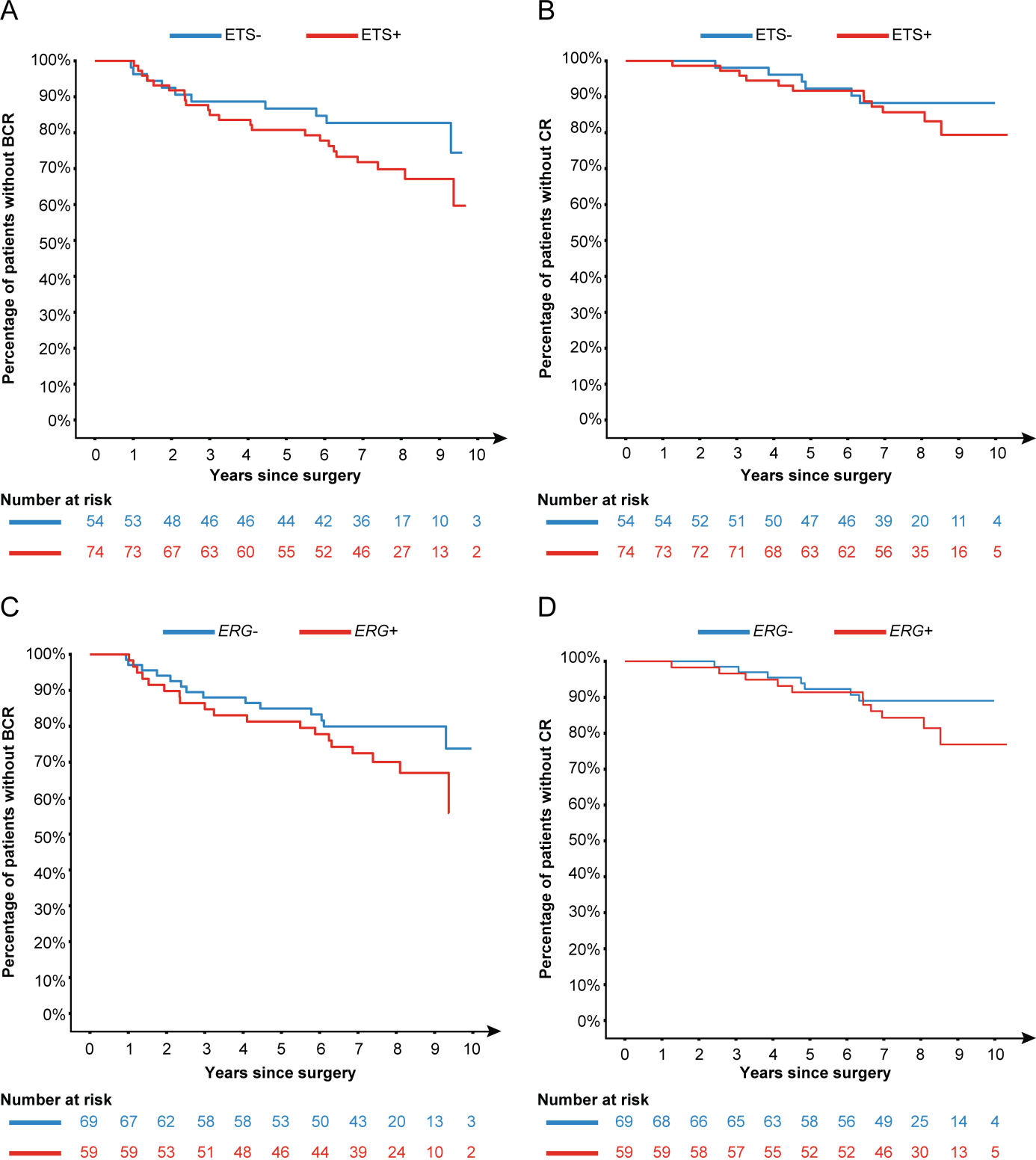


## Figure S2. RNA overexpression of ERG and additional ETS genes combined and their association with biochemical and clinical recurrence. Patients with persistently elevated PSA levels and/or who received adjuvant treatment post-radical prostatectomy were excluded from analyses (N=17). The Kaplan-Meier curves were truncated when the number at risk within a group was less than five. (A–B) Fraction of patients without (A) biochemical recurrence (log-rank test, p=0.1) or (B) clinical recurrence (log-rank test, p=0.5), stratified by ETS status in malignant samples. (C–D) Fraction of patients without (C) biochemical recurrence (log-rank test, p=0.2) or (D) clinical recurrence (log-rank test, p=0.3), stratified by *ERG* RNA overexpression status in malignant samples. BCR, biochemical recurrence; CR, clinical recurrence; ETS-, ETS-negative; ETS+, ETS-positive; *ERG-*, no *ERG* RNA overexpression; *ERG+*, *ERG* RNA overexpression; PSA, prostate-specific antigen.

##

## Table S1. REMARK checklist (Reporting recommendations for tumor marker prognostic studies) [1].

| **Item to be reported** | **Page no.** |
| --- | --- |
| **INTRODUCTION** |  |
| State the marker examined, the study objectives, and any pre-specified hypotheses. | 2 |
| **MATERIALS AND METHODS** |  |
| *Patients* |  |
| Describe the characteristics (*e.g.*, disease stage or co-morbidities) of the study patients, including their source and inclusion and exclusion criteria. | 2, Table 1, Fig. S1, Table S2, Table S6 |
| Describe treatments received and how chosen (*e.g.*, randomized or rule-based). | 2 |
| *Specimen characteristics* |  |
| Describe type of biological material used (including control samples) and methods of preservation and storage. | 2–3, Table S3, Table S5 |
| *Assay methods* |  |
| Specify the assay method used and provide (or reference) a detailed protocol, including specific reagents or kits used, quality control procedures, reproducibility assessments, quantitation methods, and scoring and reporting protocols. Specify whether and how assays were performed blinded to the study endpoint. | 3–4 |
| *Study design* |  |
| State the method of case selection, including whether prospective or retrospective and whether stratification or matching (*e.g.*, by stage of disease or age) was used. Specify the time period from which cases were taken, the end of the follow-up period, and the median follow-up time. | 2–3, Fig. S1 |
| Precisely define all clinical endpoints examined. | 2–4 |
| List all candidate variables initially examined or considered for inclusion in models. | Table S4, Table S7 |
| Give rationale for sample size; if the study was designed to detect a specified effect size, give the target power and effect size. | 2 |
| *Statistical analysis methods* |  |
| Specify all statistical methods, including details of any variable selection procedures and other model-building issues, how model assumptions were verified, and how missing data were handled. | 4, Table 2, Table S4, Table S7 |
| Clarify how marker values were handled in the analyses; if relevant, describe methods used for cutpoint determination. | 3–4 |
| **RESULTS** |  |
| *Data* |  |
| Describe the flow of patients through the study, including the number of patients included in each stage of the analysis (a diagram may be helpful) and reasons for dropout. Specifically, both overall and for each subgroup extensively examined report the numbers of patients and the number of events. | 2, 4, 6, Fig. 2, Table 2, Fig. S1 |
| Report distributions of basic demographic characteristics (at least age and sex), standard (disease-specific) prognostic variables, and tumor marker, including numbers of missing values. | Table 1, Table S2, Table S6 |
| *Analysis and presentation* |  |
| Show the relation of the marker to standard prognostic variables. | Table 1, Table 2, Table S4, Table S6, Table S7 |
| Present univariable analyses showing the relation between the marker and outcome, with the estimated effect (*e.g.*, hazard ratio and survival probability). Preferably provide similar analyses for all other variables being analyzed. For the effect of a tumor marker on a time-to-event outcome, a Kaplan-Meier plot is recommended. | Fig. 2, Fig. S2, Table S4, Table S7 |
| For key multivariable analyses, report estimated effects (*e.g.*, hazard ratio) with confidence intervals for the marker and, at least for the final model, all other variables in the model. | Table 2 |
| Among reported results, provide estimated effects with confidence intervals from an analysis in which the marker and standard prognostic variables are included, regardless of their statistical significance. | Table 2 |
| If done, report results of further investigations, such as checking assumptions, sensitivity analyses, and internal validation. | Table 2, Table S4, Table S7 |
| **DISCUSSION** |  |
| Interpret the results in the context of the pre-specified hypotheses and other relevant studies; include a discussion of limitations of the study. | 10–11 |
| Discuss implications for future research and clinical value. | 10–11 |

## Table S1. REMARK checklist (Reporting recommendations for tumor marker prognostic studies) [1].

1. McShane, L.M., D.G. Altman, W. Sauerbrei, S.E. Taube, M. Gion, and G.M. Clark, (2005). Reporting recommendations for tumor marker prognostic studies (REMARK)*.* J Natl Cancer Inst. **97**(16), 1180-1184.

|  | **Univariable Cox regression analysis** | | | |
| --- | --- | --- | --- | --- |
| **Covariable** | **Biochemical recurrence** | | **Clinical recurrence** | |
|  | **HR (95% CI)** | **p** | **HR (95% CI)** | **p** |
| **Preoperative PSA (continuous)** | 1.02 (1.01–1.03) | 0.003* | 1.01 (1.00–1.03) | 0.08 |
| **ERG protein expression** |  |  |  |  |
| *Negative* | 1.00 (reference) |  | 1.00 (reference) |  |
| *Positiv*e | 1.45 (0.93–2.24) | 0.1 | 2.35 (1.23–4.48) | 0.01* |
| **Grade Group for RP-specimen** |  |  |  |  |
| *1–2* | 1.00 (reference) |  | 1.00 (reference) |  |
| *3* | 4.30 (2.42–7.64) | <0.001* | 5.56 (2.34–13.2) | <0.001* |
| *4–5* | 7.27 (4.07–13.0) | <0.001* | 8.94 (3.75–21.3) | <0.001* |
| **pT-stage** |  |  |  |  |
| *pT2* | 1.00 (reference) |  | 1.00 (reference) |  |
| *pT3a* | 3.45 (1.96–6.09) | <0.001* | 3.76 (1.63–8.67) | 0.002* |
| *pT3b* | 9.63 (4.94–18.8) | <0.001* | 11.2 (4.39–28.4) | <0.001* |
| **pN-stage** |  |  |  |  |
| *pN0* | 1.00 (reference) |  | 1.00 (reference) |  |
| *pN1* | 5.52 (2.25–13.6) | <0.001* | 2.80 (0.80–9.78) | 0.1 |
| *pNX* | 0.61 (0.38–0.98) | 0.04* | 0.56 (0.30–1.07) | 0.08 |
| **Surgical margins** |  |  |  |  |
| *Negative* | 1.00 (reference) |  | 1.00 (reference) |  |
| *Positive* | 3.36 (1.97–5.74) | <0.001* | 2.00 (0.89–4.48) | 0.09 |

## Table S4. Univariable Cox regression analysis of ERG protein expression with biochemical recurrence and clinical recurrence as endpoints. Patients with persistently elevated PSA levels and/or who received adjuvant treatment post-radical prostatectomy were excluded from analyses (N=63). The proportional hazards assumption was met for all analyses. One patient was excluded for analysis of preoperative PSA as there was no information about preoperative PSA level. Asterisks (*) indicate statistical significance, p<0.05. CI, confidence interval; HR, hazard ratio; pN-stage, pathological lymph node stage; PSA, prostate-specific antigen; pT-stage, pathological tumor stage; RP, radical prostatectomy.

##

| **Characteristic** | **ETS-positive**  (N=85; 59%) | **ETS-negative**  (N=60; 41%) | **p** |
| --- | --- | --- | --- |
| **Age at time of surgery, median (IQR)** | 62 (58–66) | 66 (61–68) | 0.01* |
| **Preoperative PSA, median (IQR)** | 10.0 (7.9­–17.5) | 11.0 (8.2–14.0) | 0.7 |
| **Grade Group for RP-specimen, N (%)** |  |  | 0.06 |
| *1*–*2* | 35 (41) | 24 (40) |  |
| *3* | 19 (22) | 23 (38) |  |
| *4*–*5* | 31 (36) | 13 (22) |  |
| **Multifocal cancer, N (%)** | 56 (66) | 49 (82) | 0.06 |
| **Positive surgical margins, N (%)** | 14 (16) | 14 (23) | 0.4 |
| **pT-stage, N (%)** |  |  | 0.03* |
| *pT2* | 20 (24) | 26 (43) |  |
| *pT3a* | 48 (56) | 26 (43) |  |
| *pT3b* | 17 (20) | 7 (12) |  |
| *Missing* | 0 (0) | 1 (2) |  |
| **pN-stage, N (%)** |  |  | 0.1^a,b^ |
| *pN0* | 25 (29) | 18 (30) |  |
| *pN1* | 8 (9) | 1 (2) |  |
| *pNX* | 52 (61) | 41 (68) |  |
| **Cribriform pattern, N (%)** | 55 (65) | 32 (53) | 0.2 |
| **Reactive stroma, N (%)** | 46 (54) | 16 (27) | 0.002* |
| **Minor high-grade pattern 5, N (%)** |  |  | 0.4^c^ |
| *Present* | 13 (15) | 12 (20) |  |
| *Absent* | 40 (47) | 32 (53) |  |
| *Not applicable* | 32 (38) | 16 (27) |  |
| **Persistent PSA after RP, N (%)** | 7 (8) | 3 (5) | 0.5^b^ |

## Table S6. Clinicopathological characteristics of the patient subcohort analyzed on the RNA level, stratified by ETS status. The χ^2^ test of independence or Fishers exact test was used for categorical variables. The Wilcoxon rank-sum test was used for continuous variables. Asterisks (*) indicate statistical significance, p<0.05. ^a^pN0 vs.pN1; ^b^Fisher’s exact test used; ^c^Present vs. absent. IQR, interquartile range; N, number of patients; pN-stage, pathological lymph node stage; PSA, prostate-specific antigen; pT-stage, pathological tumor stage; RP, radical prostatectomy.

|  | **Univariable Cox regression analyses** | | | |
| --- | --- | --- | --- | --- |
| **Covariable** | **Biochemical recurrence**  (N=128; number of events=33) | | **Clinical recurrence**  (N=128; number of events=20) | |
|  | **HR (95% CI)** | **p** | **HR (95% CI)** | **p** |
| **Preoperative PSA (continuous)** | 1.01 (1.00–1.03) | 0.05 | 1.02 (1.00–1.04) | 0.1 |
| **ETS expression** |  |  |  |  |
| *Negative* | 1.00 (reference) |  | 1.00 (reference) |  |
| *Positive* | 1.75 (0.83–3.69) | 0.1 | 1.23 (0.49–3.14) | 0.7 |
| **Grade Group for RP-specimen** |  |  |  |  |
| *1*–*2* | 1.00 (reference) |  | 1.00 (reference) |  |
| *3* | 3.93 (1.49–10.4) | 0.006* | 3.02 (0.88–10.3) | 0.08 |
| *4*–*5* | 5.70 (2.17–15.0) | <0.001* | 4.16 (1.25–13.8) | 0.02* |
| **pT-stage** |  |  |  |  |
| *pT2* | 1.00 (reference) |  | 1.00 (reference) |  |
| *pT3a* | 9.99 (2.34–42.7) | 0.002* | 9.34 (1.21–72.0) | 0.03* |
| *pT3b* | 17.4 (3.70–81.4) | <0.001* | 19.3 (2.32–160.7) | 0.006* |
| **pN-stage** |  |  |  |  |
| *pN0* | 1.00 (reference) |  | 1.00 (reference) |  |
| *pN1* | 6.33 (1.93–20.8) | 0.002* | 1.50 (0.18–12.2) | 0.7 |
| *pNX* | 0.79 (0.37–1.70) | 0.6 | 0.62 (0.24–1.60) | 0.3 |
| **Surgical margins** |  |  |  |  |
| *Negative* | 1.00 (reference) |  | 1.00 (reference) |  |
| *Positive* | 3.63 (1.62–8.14) | 0.002* | 3.37 (1.20–9.46) | 0.02* |

## Table S7. Univariable Cox regression analysis of ETS RNA overexpression with biochemical recurrence and clinical recurrence as endpoints. Patients with persistently elevated PSA levels and/or who received adjuvant treatment post-radical prostatectomy were excluded from analyses (N=17). The proportional hazards assumption was met for all analyses except pathological node stage when using biochemical recurrence as an endpoint. Asterisks (*) indicate statistical significance, p<0.05. CI, confidence interval; HR, hazard ratio; N, number of patients; number of events, number of patients experiencing either biochemical or clinical recurrence; pN-stage, pathological lymph node stage; PSA, prostate specific antigen; pT-stage, pathological tumor stage; RP, radical prostatectomy.
